# Cavernous Hemangioma of the Cerebellopontine Angle With Remarkable Enlargement of the Internal Auditory Canal in a Child

**DOI:** 10.7759/cureus.100643

**Published:** 2026-01-02

**Authors:** Genki Honda, Hiroyuki Mishima, Jyunichi Ayabe, Taisuke Kawasaki, Yuuki Sagehashi, Youtaro Okazaki, Yoshihide Tanaka

**Affiliations:** 1 Department of Neurosurgery, Yokosuka Kyosai Hospital, Yokosuka, JPN; 2 Department of Neurosurgery, Yokohama City University Graduate School of Medicine, Yokohama, JPN

**Keywords:** cavernous hemangioma, cerebellopontine angle, child, internal auditory canal, tumor resection, vestibular schwannoma

## Abstract

Cavernous hemangioma (CH) is a benign vascular malformation. Those arising in the central nervous system commonly occur in the supratentorial region or brainstem, but those originating from cranial nerves are rare. The case was an 11-year-old girl. Presenting with left sensorineural hearing loss and unsteadiness, she was found to have a tumorous lesion in the left cerebellopontine angle, accompanied by marked enlargement of the internal auditory canal. The left vestibular schwannoma was suspected, and tumor resection was performed. However, the intraoperative findings differed from this expectation, and only partial resection was achieved. Pathological examination confirmed the diagnosis of CH. Subsequently, the residual lesion regrew to the point of compressing the brainstem, necessitating total resection after facial nerve transection. Retrospectively, the imaging findings were atypical for vestibular schwannoma. Had CH been considered in the differential diagnosis from the outset, preparation for a single-stage operation might have been possible.

## Introduction

Cavernous hemangioma (CH) is a benign vascular malformation. Those occurring in the central nervous system frequently arise in the supratentorial region or brainstem, but those arising from cranial nerves are rare [1,2]. While many CHs are asymptomatic and can be monitored, surgical resection is recommended for symptomatic lesions in easily accessible locations or those causing refractory epilepsy [3].

When a tumor in the cerebellopontine angle is identified with enlargement of the internal auditory canal, the primary differential diagnosis to consider is vestibular schwannoma [4]. On the other hand, when lesions within the internal auditory canal are accompanied by facial nerve palsy, conditions other than vestibular schwannoma must be considered, since vestibular schwannoma rarely presents with facial nerve palsy [5]. 

We report a case where we performed surgery on a lesion suspected to be a left vestibular schwannoma in a child with marked internal auditory canal enlargement. The lesion was found to be a CH. Although we prioritized facial nerve preservation and performed only partial resection, the residual tumor grew, necessitating total resection with facial nerve resection and reconstruction.

## Case presentation

Initial surgical course

Present Illness

**Figure 1:**
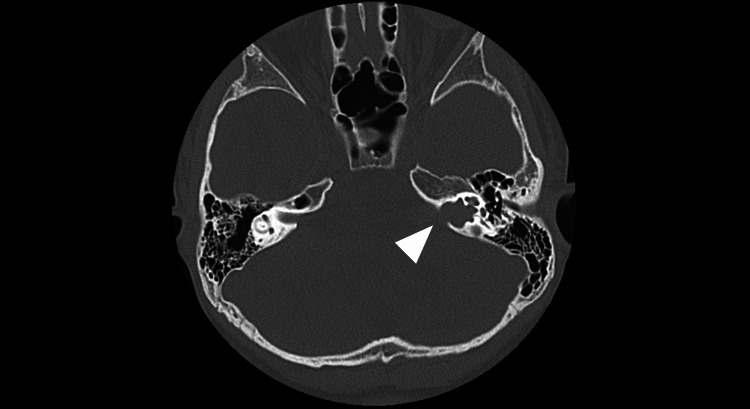
Preoperative CT scan of the skull base (bone window) Significant enlargement of the left internal auditory canal was observed (white arrowhead).

An 11-year-old female patient was referred to our otolaryngology department with chief complaints of left sensorineural hearing loss and unsteadiness. The primary symptom was progressive hearing loss in the left ear that had been developing for several months prior to referral to our hospital. The dizziness was very mild and was not objectively significant. Audiometric testing revealed decreased hearing in the left ear, and nystagmus was also induced. Head computed tomography (CT) revealed enlargement of the left internal auditory canal (Figure 1). 

**Figure 2:**
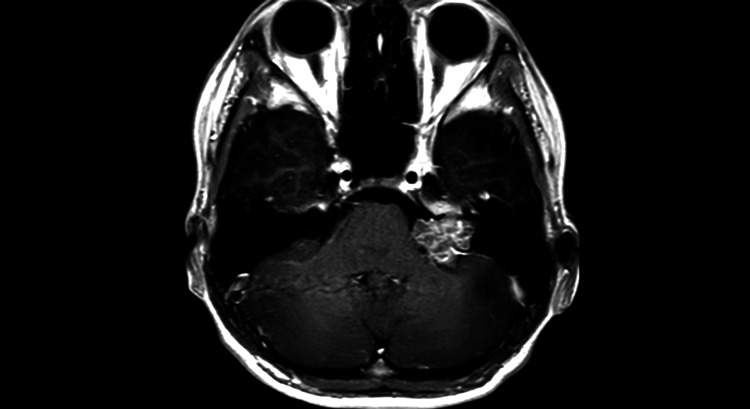
Preoperative MRI contrast-enhanced T1-weighted image A tumor shadow measuring 2.5 cm in the longest dimension was noted. It demonstrated heterogeneous contrast enhancement.

**Figure 3:**
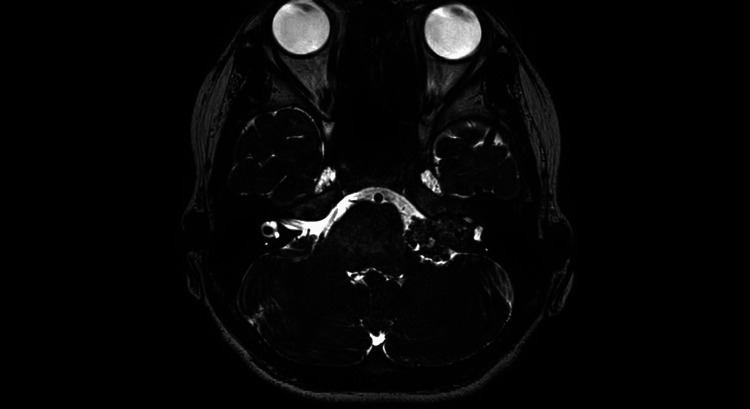
Preoperative MRI heavy T2-weighted image It mildly displaced the brainstem and cerebellum and filled the internal auditory canal.

Further examination with magnetic resonance imaging (MRI) identified a tumorous lesion in the left cerebellopontine angle, leading to referral to our department. Physical examination at the time of presentation showed no paralysis in the limbs and no obvious facial motor paralysis, but left hearing loss was present. A head contrast-enhanced MRI revealed a tumor shadow with a maximum diameter of 2.5 cm extending from the left internal auditory canal to the cerebellopontine angle. The lesion showed a flow void on T1-weighted images and heterogeneous contrast enhancement. The tumor caused mild brainstem compression (Figures 2, 3). Cerebral angiography did not reveal any obvious tumor enhancement or other vascular malformations.

Based on the above findings, the primary differential diagnosis was considered to be a left vestibular schwannoma. Given the large tumor size and resulting brainstem compression, which was expected to impact long-term prognosis, a decision was made to proceed with surgery. 

Surgical Findings

**Figure 4:**
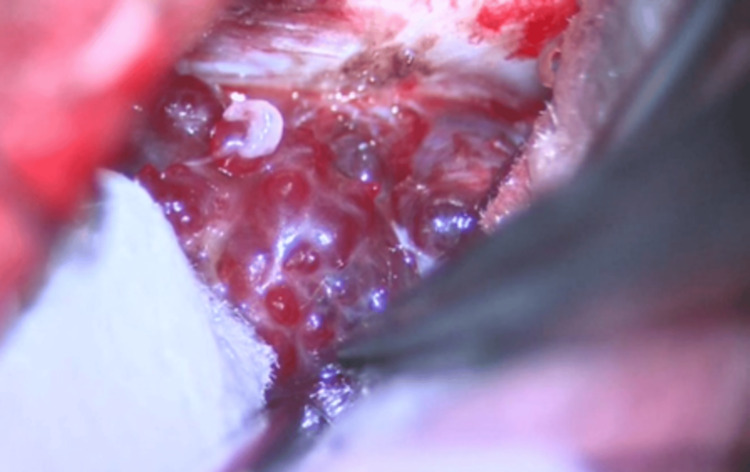
Intraoperative findings The tumor consisted primarily of a hematoma-like cyst.

A nerve integrity monitor (NIM) (Medtronic's NIM System 3.0 (Medtronic, MN, USA) with four channels: the frontalis muscle, orbicularis oculi muscle, orbicularis oris muscle, and mentalis muscle) was installed, and a left lateral retrosigmoid craniotomy was performed. Microscopically, the tumor was identified in the lateral cerebellomedullary cistern. The lesion primarily consisted of a hematoma-like cyst, presenting findings distinct from a typical vestibular schwannoma (Figure 4). Intraoperative pathology was performed, but despite multiple submissions, only connective tissue with inflammatory cell infiltration was detected; no tumor tissue was identified. The tumor was also strongly adherent to the surrounding brainstem. Conventional resection for internal decompression was difficult, necessitating removal via bipolar cautery and reduction. The facial nerve traversed through the tumor, and a response was observed at most points on the tumor surface with a 0.1 mA stimulus from the NIM. The bone of the posterior wall of the internal auditory canal within the petrous bone was removed, and the tumor within the canal was resected as completely as possible, leaving behind the tumor adherent to the facial nerve.

Postoperative Course

**Figure 5:**
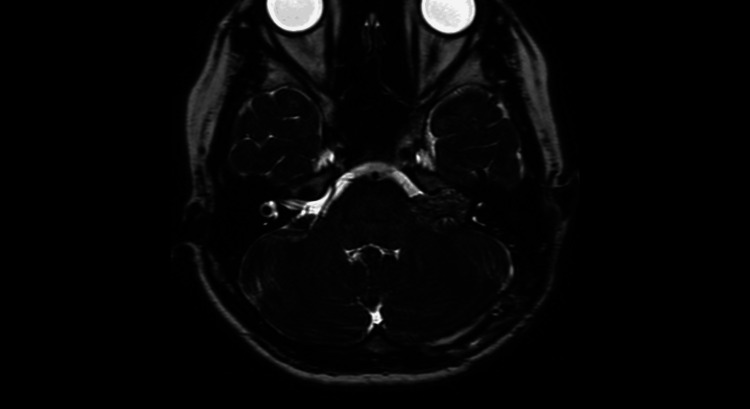
Postoperative MRI T2-weighted image Residual tumor was noted, but the compressive effect on the brainstem and cerebellum had been reduced.

No new neurological deficits, including obvious facial motor paralysis, developed postoperatively. Hearing had deteriorated significantly just before surgery and showed no improvement afterward. There was also no change in dizziness compared to before surgery. Although a residual tumor was present within the internal auditory canal on postoperative head CT and MRI, the compressive effect on the brainstem and cerebellum was relieved (Figure 5). The patient progressed favorably and was discharged home on the eighth postoperative day. 

Pathological Findings

**Figure 6:**
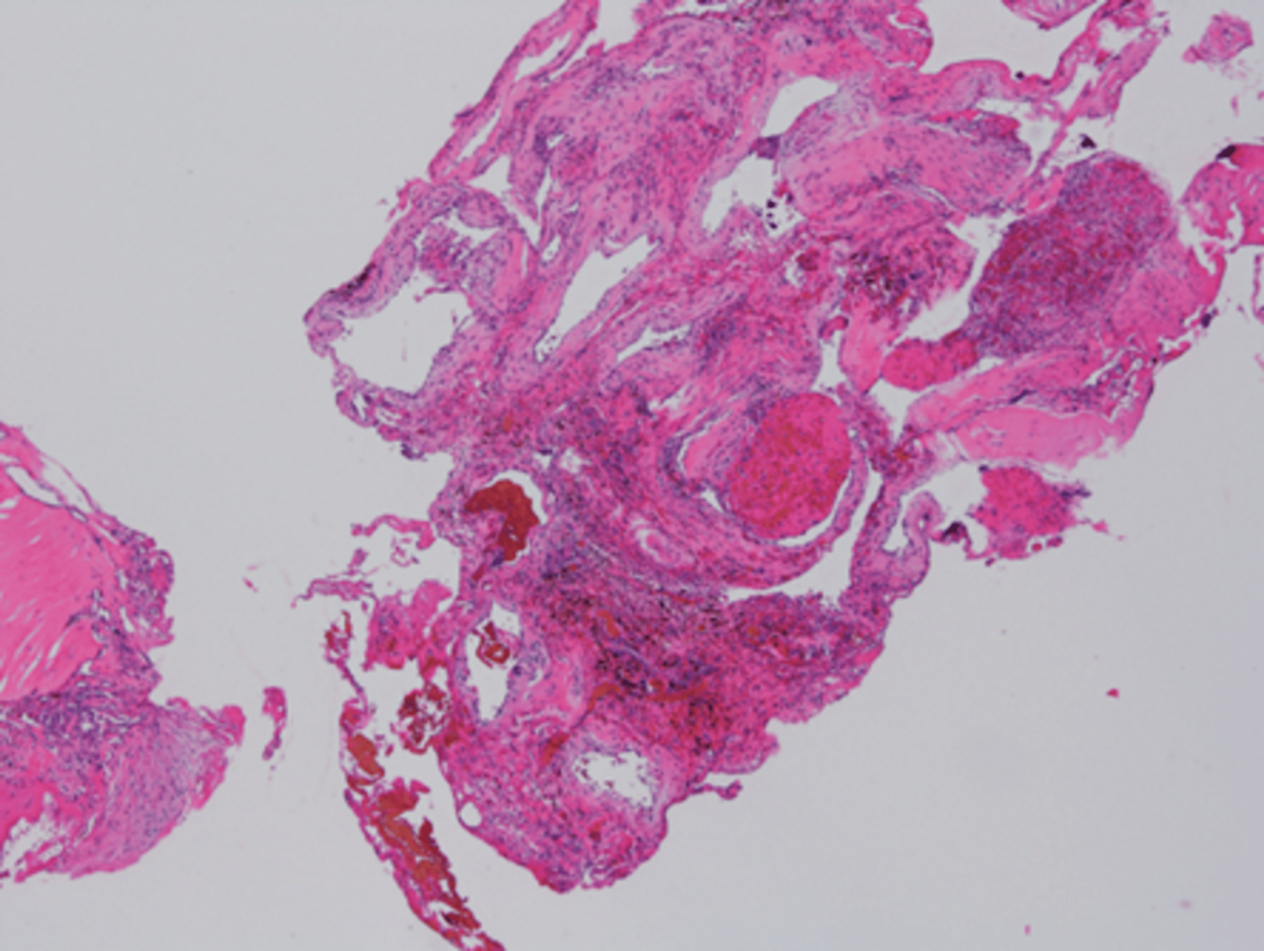
Hematoxylin and eosin-stained pathological specimen The specimen showed proliferative changes in dilated vessels, with organically formed thrombi and vitrified thrombi visible within the vessels.

Proliferation of dilated vessels was observed, along with organically transformed thrombi and vitrified thrombi within the vessels (Figure 6). Immunohistochemical staining revealed CD31(+), SMA(+), and D2-40(-). A diagnosis of CH was made.

Course leading to reoperation

**Figure 7:**
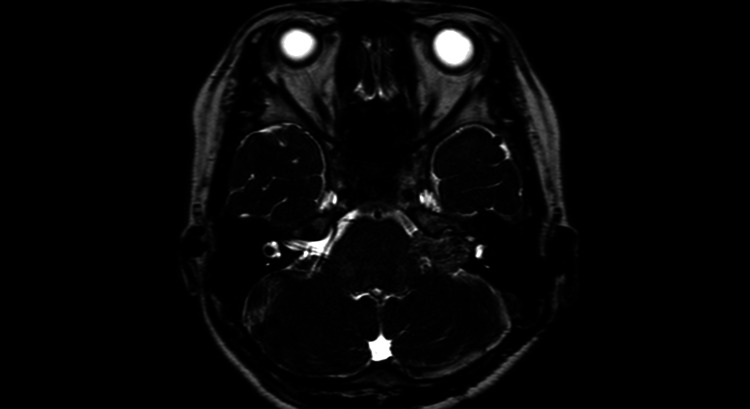
One year postoperative MRI heavy T2-weighted image

**Figure 8:**
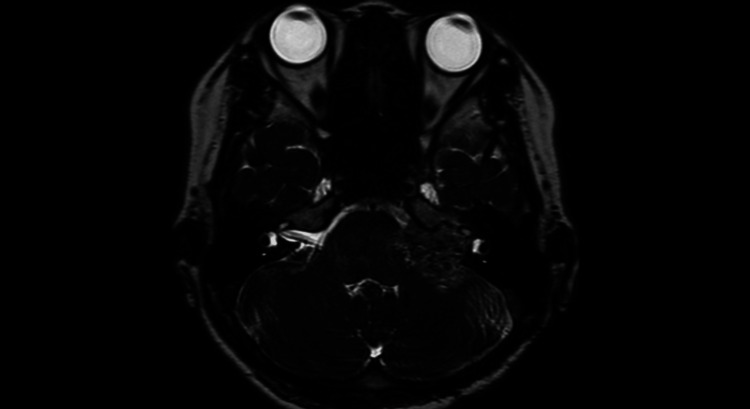
A 2.5 years postoperative MRI heavy T2-weighted image

Following discharge, imaging follow-up was performed in the outpatient setting. Symptoms remained stable, but MRI revealed a progressive increase in residual tumor volume over time. The progression of the tumor's maximum diameter, measured from the peripheral inner ear canal toward the pons, was as follows: preoperative: 22.8 mm; one year postoperative: 24.7 mm; 1.5 years postoperative: 28.5 mm (Figure 7). During the follow-up period, radiation therapy was considered as an option. However, since the tumor was suspected to have originated in the facial nerve, the risk of nerve damage from radiation exposure was deemed high. Therefore, at that time, the decision was made to proceed with follow-up imaging studies. At the 2.5-year postoperative MRI, brainstem compression had worsened; the tumor's maximum diameter was 31.4 mm (Figure 8), and a physical examination at the same time period also noted the onset of left facial paralysis. Given the worsening trend in both imaging and symptoms, which was considered likely to impact long-term prognosis, a decision was made to perform a second open craniotomy for tumor resection two years and seven months after the initial surgery. At that time, based on findings from the previous surgery, it was explained repeatedly that facial nerve preservation would be difficult and facial nerve palsy was highly likely to occur before proceeding with the operation.

Surgical Findings

**Figure 9:**
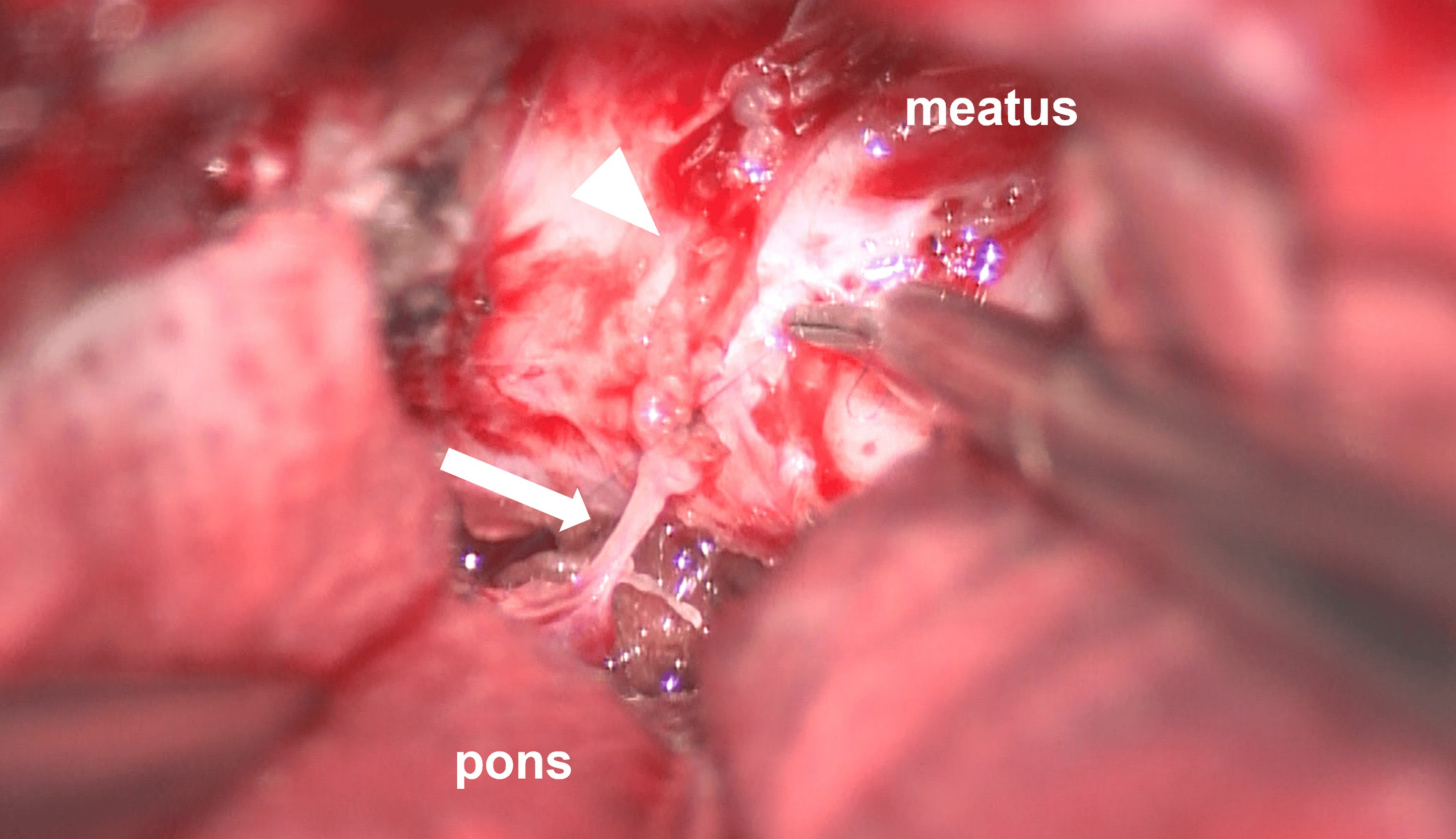
Suturing the facial nerve The brainstem stump (white arrow) and the internal auditory canal stump (white arrowhead) of the facial nerve were sutured using 10-0 nylon.

The procedure was performed under the same patient position, skin incision, and facial nerve monitoring as the previous surgery. Due to significant adhesions with surrounding structures, endoscopic decompression using an ultrasonic scalpel was difficult. Therefore, as in the previous surgery, resection was performed by repeatedly applying bipolar cautery. As tumor removal progressed, the amplitude on facial nerve monitoring gradually decreased. The facial nerve branched within the tumor, making it difficult to follow its course. The tumor was dissected away from the abducens nerve and the trigeminal nerve. The posterior wall of the internal auditory canal within the petrous bone was removed, and the remaining hemangioma within the internal auditory canal was completely excised. The facial nerve was transected during this excision. The facial nerve stump within the internal auditory canal was sutured to the brainstem stump using a single 10-0 nylon suture; in reality, only partial nerve fibers remained at both ends (Figure 9). The exposed internal auditory canal was closed using bone wax, muscle grafts, and DuraGen® (Integra LifeSciences, NJ, USA).

Postoperative Course

**Figure 10:**
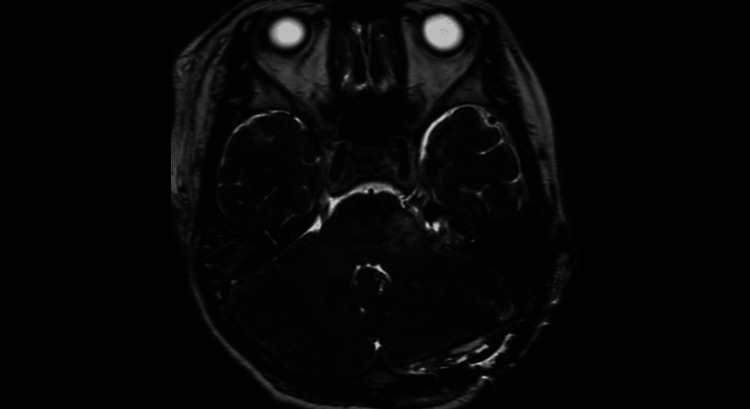
Immediately after reoperation, MRI T2-weighted image The tumor had been completely removed, and the compression on the brainstem had been relieved.

**Figure 11:**
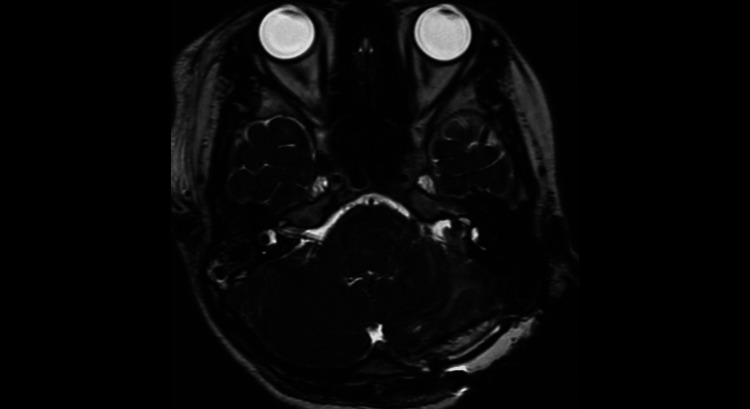
Immediately after reoperation, MRI T2-weighted image All tumors within the internal auditory canal had been completely removed.

Left facial motor function showed paresis of the corner of the mouth and mild paresis of eyelid closure, graded as House-Brackmann (H-B) Grade V. The left facial paralysis gradually improved, and the left eye regained the ability to close. Postoperative MRI confirmed complete resection of the lesion (Figures 10, 11). The patient was ultimately discharged on postoperative day 16, with a discharge H-B Grade of III. 

## Discussion

CH is a benign vascular malformation characterized histologically by clusters of dilated blood vessels sharing a common wall. Its prevalence is relatively high at approximately 0.5% [6], accounting for 10-15% of central nervous system vascular malformations [7]. Around 25-50% are asymptomatic [8], allowing for observation in such cases. However, when symptomatic, they may cause seizures, headaches associated with bleeding, and neurological deficits. Historical literature reports that cases presenting with cranial nerve deficits often involved hemorrhage in the brainstem region [9]. Furthermore, in CH, where symptoms progressively worsen over time, enlargement of the hematoma cavity is generally considered the cause [10]. CH arising from cranial nerve development is extremely rare, with limited case reports [1,2].

In this case, CT revealed enlargement of the internal auditory canal. Since the facial nerve stump was identified within the canal after CH resection, it is presumed that the tumor originated within the canal and subsequently extended to the cerebellopontine angle. Cerebral nerve CHs are thought to arise from the vascular system of the epineurium or the vascular plexus surrounding the Scarpa ganglion, causing local nerve compression and resulting in neurological deficits [11].

Reports of surgical intervention for CHs arising in the internal auditory canal have been documented [12,13], but no reports of residual tumor recurrence were found within the scope of our search. In this case, vestibular schwannoma was initially suspected. We aimed for maximal tumor resection under NIM monitoring while preserving the facial nerve. However, the intraoperative findings differed from those of a vestibular schwannoma. Furthermore, NIM was positive in most areas of the lesion. Therefore, considering functional preservation, we had no choice but to leave a significant portion of the tumor attached to the facial nerve. During postoperative follow-up, the residual lesion progressively enlarged, causing facial nerve palsy and worsening brainstem compression. Furthermore, the pattern of enlargement was not due to internal hemorrhage or cystic expansion within the lesion, but rather the tumor itself was solidly increasing in size. Given this course, from the perspective of ensuring a favorable prognosis, it was determined that complete resection of the lesion, necessitating facial nerve transection, was unavoidable.

CH is a benign disease, and we often encounter cases that progress without requiring surgery, managed solely by imaging follow-up. However, cases like the present one, or those with a solid mass several centimeters in size, suggestive of CH enlarging over time, remind us that CH is a disease capable of tumor-like growth. Factors associated with CH enlargement may include high expression of Ki-67 and bcl-2 within the tumor itself, as well as high expression of growth factors such as TGFβ, PDGF, and tenascin in the surrounding brain parenchyma [14]. Predicting enlargement based on imaging findings alone is difficult, however, and imaging follow-up should be conducted with the possibility of enlargement in mind.

According to Li et al., CHs arising from cranial nerve development are classified into three types: intraneural, perineural, and extraneural. The intraneural type accounts for 51.4% of cases and is associated with a significantly higher risk of postoperative neurological deterioration compared to the other types [15]. Intraoperative findings in this case (evidence of reaction over the entire tumor surface on NIM, and the facial nerve branching and running within the tumor) suggest an intraneural type tumor. This further indicates that facial nerve preservation was difficult in this case.

CHs arising within the internal auditory canal are frequently misinterpreted as vestibular schwannomas on imaging studies, as was the case in this patient. A case series study noted that vestibular schwannomas rarely cause facial nerve deficits; therefore, when an internal auditory canal lesion presents with facial nerve deficits, CH is strongly suspected [5]. Conversely, in cases like this one, where the presentation was solely hearing loss, differentiation from vestibular schwannoma becomes challenging.

However, retrospectively reviewing this case, the finding of non-cystic, heterogeneous contrast enhancement on T1-weighted contrast-enhanced MRI is atypical for vestibular schwannoma. At that stage, the possibility of diseases other than vestibular schwannoma and the potential for intraoperative diagnosis changes should have been fully considered during surgical preparation. Furthermore, the marked enlargement of the internal auditory canal observed in a benign condition such as CH is presumed to have been caused by the high plasticity of pediatric bone.

In cases where CH was suspected from the outset, considering that no reports exist of achieving total resection while preserving facial nerve function in similar past cases, performing a single-stage total resection from the initial surgery, with the premise of sacrificing and reconstructing the facial nerve, might have avoided the burden of reoperation. When encountering future cases presenting similar imaging findings, it will be necessary to fully explain to the patient the potential difficulty of nerve preservation and reach a shared decision regarding surgery.

## Conclusions

We encountered a case where surgery was performed on a lesion suspected to be a left vestibular schwannoma with marked enlargement of the internal auditory canal. However, it was identified as a CH, and only partial resection was performed. Subsequently, the residual tumor grew, necessitating total resection with facial nerve resection and reconstruction. When a tumorous lesion is identified in the cerebellopontine angle, vestibular schwannoma is often considered a differential diagnosis. However, if imaging reveals atypical findings, it is advisable to formulate a surgical plan that also considers other possible conditions, such as CH.

Although CH is a benign tumor, some cases exhibit progressive enlargement. In such instances, thorough informed consent must be obtained from the patient, taking into account the risks of complications. Accumulation of similar cases and consideration of safer treatment methods are awaited.
